# Descemet membrane endothelial keratoplasty (DMEK) improves vision-related quality of life

**DOI:** 10.1007/s00417-022-05711-9

**Published:** 2022-05-25

**Authors:** Alexandra Gellert, Jan Darius Unterlauft, Matus Rehak, Christian Girbardt

**Affiliations:** 1 Klinik und Poliklinik für Augenheilkunde, Liebigstraße 12, 04103 Leipzig, Germany; 2 Universitätsklinik Für Augenheilkunde, Inselspital, Freiburgstrasse, 3010 Bern, Switzerland; 3 Klinik Und Poliklinik Für Augenheilkunde, Friedrichstr. 18, 35392 Gießen, Germany

**Keywords:** Descemet membrane endothelial keratoplasty, Corneal transplantation, Quality of life

## Abstract

**Purpose:**

To evaluate vision-related quality of life (VRQL) before and after Descemet membrane endothelial keratoplasty (DMEK).

**Methods:**

The study was conducted in patients with Fuchs endothelial dystrophy or pseudophakic bullous keratopathy undergoing DMEK alone or in combination with cataract surgery (triple-DMEK) between August 2019 and March 2020 at the University of Leipzig Medical Center. Best-corrected visual acuity (BCVA) was measured. Visual acuity factor (VAF) and glare factor (GF) scores were calculated using the Visual Function and Corneal Health Status Instrument questionnaire answered by patients before surgery and 6 months thereafter. Subgroup analyses were performed for DMEK versus triple-DMEK, and for first versus second eyes, in addition to correlation analyses of scores with preoperative BCVA.

**Results:**

Forty-six patients were included in this analysis. VAF score improved from 0.68 ± 0.54 to 0.02 ± 0.57 (*P* < 0.0001) and GF score improved from 0.53 ± 0.43 to -0.11 ± 0.39 (*P* < 0.0001) during follow-up. Both scores improved without significant differences after surgery in the first and in the fellow eye (*P* < 0.0001) and after DMEK and triple-DMEK (*P* < 0.0001). The improvement of scores did not correlate with preoperative BCVA (*r* = 0.06, *P* = 0.68 for VAF; *r* =  -0.09, *P* = 0.54 for GF).

**Conclusion:**

VRQL improves similarly after DMEK and triple-DMEK and between first and second operated eye. The extent of improvement is independent of the preoperative BCVA. The results of this study can be useful when planning DMEK by enabling a prediction of anticipated VRQL gain.

**Supplementary Information:**

The online version contains supplementary material available at 10.1007/s00417-022-05711-9.


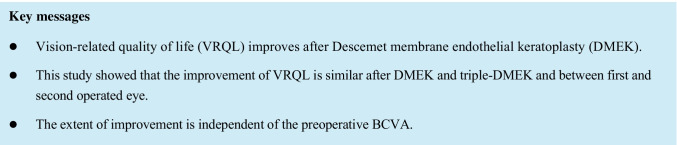


## Introduction

Measuring quality of life has become increasingly important in medicine. It has been established as a subjective criterion of therapeutic success from the patient’s perspective, and its improvement is an important therapeutic goal for the treating physician [1].

Health-associated quality of life is best evaluated using standardized instruments. Apart from generic questionnaires [2], disease-specific questionnaires are available. The “Visual Function and Corneal Health Status Instrument” (V-FUCHS) [3, 4] is specifically designed for vision-related quality of life evaluation in Fuchs endothelial dystrophy (FED).

Corneal endothelial diseases such as FED and pseudophakic bullous keratopathy (PBK) are characterized by loss of pump and/or barrier function of the endothelium, resulting in stromal and epithelial edema. The two main complaints of patients with corneal endothelial diseases are impairment of central visual acuity and glare [5, 6]. Since its introduction in 2006 by Gerrit Melles [7], Descemet membrane endothelial keratoplasty (DMEK) has become the gold standard therapy for corneal endothelial diseases [8–10]. The procedure can be performed stand-alone or in combination with phacoemulsification and intraocular lens implantation (triple-DMEK) [11]. In order to assess the optimal timing for the surgical intervention, it is important to consider the risk of subepithelial scar formation in long-standing corneal decompensation on the one hand, as well as the worldwide graft shortage with the consequence of restricted indication on the other hand. Therefore, apart from objective criteria such as visual acuity, specular microscopy, and pachymetry, it seems reasonable to also evaluate the patient’s subjective complaints and their possible improvement through DMEK surgery [12].

High subjective satisfaction and quality of life improvement have already been shown in penetrating keratoplasty (PK) and Descemet stripping automated endothelial keratoplasty (DSAEK) [13–19]. For DMEK however, only few studies have addressed the subject of quality of life changes. The instruments used were merely different versions of the National Eye Institute Visual Function Questionnaire (NEI VFQ-25 and NEI VFQ-39) as used in quality of life evaluation in chronic eye diseases in general [20–22]. In contrast, the V-FUCHS allows a specific assessment of disturbing factors in FED [4].

Our study aimed to investigate the impact of DMEK on patients’ vision-related quality of life using the V-FUCHS questionnaire. In order to facilitate patient counseling, we also looked for possible differences in quality of life changes between first and fellow eyes, as well as between DMEK alone and triple-DMEK cases. Furthermore, possible associations between quality of life changes with patient age and preoperative visual acuity were investigated.

## Materials and methods

### Study design

The study was conducted in patients receiving DMEK for FED or PBK at the University of Leipzig Medical Center between August 2019 and March 2020. The exclusion criteria were intraoperative complications influencing postoperative visual acuity and comprehension problems when answering the questionnaire that would have impeded data analysis.

### Questionnaire

The German version of the V-FUCHS instrument in its version of July 2019 [3] was used. The V-FUCHS has been specifically designed for evaluation of vision-related quality of life in FED patients and comprises 15 questions on vision in everyday life activities [4]. Eight questions on glare and intraday fluctuations result in a glare factor (GF) and seven questions on visual acuity result in a visual acuity factor (VAF). The individual questions are answered on a Likert scale by ticking one of five categories scaled ordinally (see Online Resource 1). As two of the items could be interpreted as referring to complaints when driving a car, we additionally recorded the status of whether patients drive on their own at all.

### Procedure

V-FUCHS was completed by the patients the day before DMEK surgery. Questions were read aloud for patients with insufficient visual acuity. DMEK was performed as a stand-alone procedure in pseudophakic patients or as a triple procedure when cataract was present. All surgical procedures were performed by two experienced corneal surgeons (CG, JDU). On-site graft preparation was performed using the “liquid bubble” technique [23]. Surgery was performed utilizing the “no touch” technique [24]. The questionnaire was completed again 6 months post-surgery. Best-corrected visual acuity (BCVA) was recorded during pre- and post-operative routine examinations. BCVA was assessed as a distant visual acuity test with a 5-m testing distance and best spectacle correction using Snellen charts in a chart projector.

### Statistics

Statistical analyses were performed using the Partial Credit Model. Ordinally scaled raw data corresponding to answers on the Likert scale were transformed to Logits. The Logits correspond to the underlying impediment of the patient (latent variable) on a linear scale [25]. As a result, non-dimensional pre- and post-operative values of GF and VAF scores were obtained. Data are presented as mean and standard deviation. Theoretical maximal GF score ranges from -0.74 to 1.59 and VAF score reaches from -0.73 to 1.77, the respective minimal value corresponding to complete absence of discomfort and the maximal value to the highest impediment. Subgroup analyses for pseudophakic DMEK versus triple-DMEK and for first versus second eye were performed.

BCVA measurements were converted to the logarithm of the minimum angle of resolution (logMAR).

All statistical calculations were performed using IBM SPSS Statistics 25 and Microsoft Excel 2019. Significance was tested using Student’s *t*-test for paired samples for comparison of preoperative and postoperative scores. Subgroup analysis was performed using Student’s *t*-test for independent samples and Mann–Whitney *U* test. Significance level was set at *α* = 0.05.

Correlation analyses of scores with patient age and BCVA were performed using either Pearson’s product-moment correlation or Spearman’s rank correlation when normal distribution was not present.

Planned subgroup analysis was used, so adjusting the level of significance was not necessary.

Patients who were operated on both eyes during the study were included only with data before and after the second operation in order to maintain independence of samples. Second eyes were always operated on 6 months or later after the first eye.

## Results

### Patient characteristics

The study was conducted on 49 patients. One patient did not answer the questionnaire postoperatively. From the remaining 48 patients, two were excluded: one due to comprehension problems and one due to massive dry eye syndrome subjectively impeding quality of life. Ten patients had ocular comorbidities possibly affecting BCVA, whereas 36 patients had comorbidities not affecting BCVA or no comorbidities. Four patients were operated on both eyes during the study and were included only with their second eye as described above. Patient characteristics are shown in Table 1.

**Table 1:** Baseline characteristics

	Number *n* (%)	Age (years) mean ± SD	Female *n* (%)	Car driver *n* (%)	BCVA (logMAR) preoperative mean ± SD	BCVA (logMAR) postoperative mean ± SD
Total	46 (100)	72.5 ± 7.3	27 (58.7)	36 (78.2)	0.68 ± 0.52	0.21 ± 0.24
No ocular comorbidities or not affecting BCVA^b^	36 (78.3)	72.0 ± 7.2	22 (0.6)	30 (0.8)	0.59 ± 0.45	0.14 ± 0.17
Ocular comorbidities affecting BCVA^a^	10 (21.7)	74.1 ± 7.9	5 (50.0)	6 (60.0)	0.99 ± 0.66	0.43 ± 0.32
Diagnosis						
FED	38 (82.6)	72.1 ± 7.8	22 (57.9)	29 (76.3)	0.57 ± 0.41	0.19 ± 0.23
PBK	8 (17.4)	74.0 ± 5.1	5 (62.5)	7 (87.5)	1.17 ± 0.74	0.31 ± 0.26
Operation						
DMEK	22 (47.8)	73.8 ± 5.9	14 (63.6)	17 (77.2)	0.87 ± 0.64	0.31 ± 0.30
Triple-DMEK	24 (52.2)	71.3 ± 8.1	13 (54.2)	19 (79.2)	0.50 ± 0.30	0.11 ± 0.11
Operated eye						
First eye	28 (60.9)	72.8 ± 6.9	15 (53.6)	24 (85.7)	0.77 ± 0.62	0.24 ± 0.29
Second eye	18 (39.1)	71.9 ± 8.2	12 (66.7)	12 (66.7)	0.53 ± 0.30	0.15 ± 0.11
Cataract						
Incipient	15	70.47 ± 9.0	8 (53.3)	14 (93.3)	0.47 ± 0.35	0.11 ± 0.10
Advanced	10	72.90 ± 6.32	6 (60.0)	6 (60.0)	0.52 ± 0.21	0.12 ± 0.12

*FED* Fuchs endothelial dystrophy, *PBK* pseudophakic bullous keratopathy, *SD* standard deviation, *BCVA* best corrected visual acuity

^a^Myopia, dry age-related macular degeneration, optic atrophy, diabetic retinopathy, capsular fibrosis

^b^Dermatochalasis, post laser coagulation for retinal foramen

### Scores in V-FUCHS questionnaire

VAF score preoperative mean was 0.68 (SD = 0.54) and postoperative mean was 0.02 (SD = 0.57), respectively. There was no difference between PBK and FED patients in preoperative scores (VAF score FED 0.63 ± 0.56, PBK 0.93 ± 0.36, *p* = 0.34; GF score FED 0.51 ± 0.44, PBK 0.62 ± 0.41, *p* = 0.5). Mean preoperative GF score was 0.53 (SD = 0.43) and postoperative mean was -0.11 (SD = 0.39). Postoperative values were significantly lower than preoperative values for both scores (*P* < 0.0001).

Female and male patients improved equally in both scores (difference VAF score: 0.68 ± 0.57 versus 0.64 ± 0.71, P = 0.82; difference GF score: 0.69 ± 0.47 versus 0.57 ± 0.43, *P* = 0.37).

Detailed analysis for operated eye and type of operation is given in Table 2.

**Table 2:** Subgroup analysis by operated eye and type of operation

	First eye			Second eye		
Score mean ± SD	Preoperative	Postoperative	*P*	Preoperative	Postoperative	*P*
Visual acuity factor	0.85 ± 0.42	0.13 ± 0.63	< 0.0001	0.41 ± 0.59	-0.15 ± 0.42	< 0.0001
Glare factor	0.62 ± 0.37	-0.05 ± 0.4	< 0.0001	0.38 ± 0.49	-0.21 ± 0.36	< 0.0001
	Pseudophakic DMEK			Triple-DMEK		
Score mean ± SD	Preoperative	Postoperative	*P*	Preoperative	Postoperative	*P*
Visual acuity factor	0.79 ± 0.44	0.19 ± 0.61	< 0.0001	0.59 ± 0.60	-0.13 ± 0.50	< 0.0001
Glare factor	0.63 ± 0.36	0.03 ± 0.41	< 0.0001	0.43 ± 0.47	-0.24 ± 0.33	< 0.0001

*SD* standard deviation

Both VAF and GF scores improved with each operation of the first (28 patients) and second eye (18 patients). There was no significant difference in the extent of improvement between first and second eye both in VAF (first eye: 0.72 ± 0.65; second eye: 0.57 ± 0.57; *P* = 0.42) as well as GF (first eye: 0.67 ± 0.44; second eye: 0.59 ± 0.48; *P* = 0.56).

VAF and GF scores improved in pseudophakic as well as triple-DMEK cases, without a significant difference in improvement between both groups (VAF pseudophakic DMEK: 0.60 ± 0.58, triple-DMEK: 0.72 ± 0.66, *P* = 0.53; GF pseudophakic DMEK: 0.60 ± 0.41, triple-DMEK: 0.67 ± 0.5, *P* = 0.60).

When comparing the postoperative scores, there was a significant difference between DMEK alone and triple DMEK cases in the GF score (postoperative GF score DMEK alone: 0.03 ± 0.41; postoperative GF score triple DMEK: -0.24 ± 0.33; *P* = 0.02), but not in the VAF score (postoperative VAF score DMEK alone: 0.19 ± 0.61; postoperative VAF score triple DMEK: -0.13 ± 0.5; *P* = 0.06).

### Correlations with age and preoperative BCVA

VAF gain (*r* =  -0.03, *P* = 0.87) and GF score gain (*r* =  -0.14, *P* = 0.35) were independent of patient age.

There was a moderate correlation between postoperative BCVA and GF score (*r* = 0.57, *P* < 0.0001) and a weak correlation between postoperative BCVA and postoperative VAF score (*r* = 0.41, *P* = 0.004).

There was no significant correlation between preoperative BCVA and VAF change (*r* = 0.06, *P* = 0.68) or GF score change (*r* =  − 0.09, *P* = 0.54) during follow-up.

## Discussion

Before the development of posterior lamellar keratoplasty techniques, indication for PK in corneal endothelial disorders was only given in cases of significant corneal decompensation with edema, pronounced visual acuity loss, and pain. With the success of DMEK and DSAEK, this concept has changed significantly, and quality of life aspects have come to the fore. DMEK can be indicated when subjective complaints from glare and everyday life limitations are perceived, even if standard visual acuity examination still shows comparatively good results [26, 27]. These subjective limitations in vision can be assessed using the V-FUCHS questionnaire at different stages of FED. Until now, no results of V-FUCHS testing before and after intervention have been published [12].

Both VAF and GF scores improved from preoperative to postoperative values in our study, clearly demonstrating a reduction in difficulties due to vision in everyday life and consequently an improvement in vision-related quality of life. This is in accordance with results reported by Dunker et al. [20], who showed improvement of vision-related quality of life 3 months after DMEK using NEI VFQ-25. Bayyoud et al. [21] showed improvement in vision-related difficulties in everyday life and driving using a modified version of the NEI VFQ-25. Secondary analysis of the “Descemet Endothelial Thickness Comparison Trial” [23] measured vision-related quality of life after DMEK using NEI VFQ-39 and showed comparable improvement 3 and 12 months after surgery. However, in comparison to the V-FUCHS, these instruments focus more on general health status, vision in general, eye pain, and psychosocial components. The V-FUCHS item profile however is specifically designed for the evaluation of endothelial diseases and thus offers a more specific instrument for evaluation of vision-related quality of life after DMEK.

Our study showed no differences in vision-related quality of life gain between the operation on first versus second eye. This is an extension to the results of Siggel et al. who showed that sequential bilateral DMEK led to comparable amelioration of BCVA, endothelial cell density, and corneal thickness [28]. Thus, physicians counseling patients after successful operation on one eye can give their patients the expectation of an equal quality of life gain after surgery of the fellow eye.

DMEK and triple DMEK patients showed similar improvements in quality of life in our study. This is in contrast to a study published by Trousdale et al. [19], who examined quality of life in FED patients undergoing PK, deep lamellar endothelial keratoplasty, and Descemet-stripping endothelial keratoplasty (DSEK) using NEI VFQ-25 and showed that greater improvement was achieved when corneal graft and cataract surgery were combined. This discrepancy in results could be due to the use of a different questionnaire that included other items such as eye pain and psychological impairment as indicators for quality of life. It is possible that the V-FUCHS, with its design specifically for complaints from endothelial decompensation, does not cover the presence of a concomitant cataract. Furthermore, the aforementioned study only included FED patients. It is possible that in our study, patients receiving DMEK showed comparable baseline characteristics, in the sense that PBK patients might receive surgery at a comparable stage of quality of life impairment as patients with FED and cataract combined.

We found quality of life improvements to be independent of preoperative BCVA, meaning that patients with lower as well as higher preoperative visual acuity benefit equally from DMEK concerning quality of life.

Concerning associations between BCVA and quality of life, existing studies on DSAEK and PK have shown no correlation between BCVA improvement and quality of life after surgery [16, 18]. Our results showed a correlation between postoperative BCVA and V-FUCHS-Scores, i.e., as expected, higher visual acuity values were associated with less quality of life impediments. However, as correlation was low, we conclude that BCVA as measured after DMEK makes up for only one aspect of vision-related quality of life. This is supported by results from studies showing improvement in contrast sensitivity and color vision after DMEK [21, 29, 30].

We did not find any gender-specific differences in quality of life changes after surgery. There are no comparable data in the literature concerning DMEK. The “Corneal Transplant Epidemiological Study” [15] showed lower quality of life scores for female patients compared to male patients for PK and anterior lamellar keratoplasty. However, apart from the other types of keratoplasty examined, quality of life scores were assessed using other items relating to physical and mental health.

Patient age showed no correlation with quality of life improvement in our study. Again, there are no comparable results on this topic concerning DMEK. Diverging results have been found in other types of keratoplasty: Mendes et al. [16] found an association between quality of life improvement and younger patient age in PK. Their study however had a broad spectrum of underlying diagnoses and a wide age spectrum. Puri et al. [17], on the other hand, found an association between quality of life improvement and older patient age in PK, DSAEK, and keratoprosthesis. Direct comparison to our data is not possible due to heterogeneous study groups.

Our study has several limitations. Since the final common pathway of corneal decompensation and complaints are comparable between different types of endothelial disorders, we also used V-FUCHS for PBK patients with their typically more severely decompensated corneas, although it had originally been designed for FED patients only. Possible underlying differences in morphological changes of the cornea in this mixed study-population of FED and PBK might have impeded our analysis. The triple-DMEK consisted purely of FED patients, whereas all PBK patients naturally all appear in the DMEK only group. We did not perform further subgroup analysis due to the rather small sample size. Further study with bigger case numbers might find differences between groups and elucidate the fact if some subgroups of patients profit more in terms of quality of life than others. In our study, patients with and without ocular comorbidities were included which might have impeded the results. We were aware of that potential problem, but we sought to investigate quality of life improvements via DMEK in a real-world setting, where DMEK patients present to our clinic with a broad spectrum of ocular comorbidities. Limitations within V-FUCHS itself might be found in items 2 and 3, where complaints in the morning are explicitly assessed. Some of our patients also reported complaints following an afternoon nap, thus impeding a conclusive answer. Finally, there might be a bias because the Partial Credit Model evaluation tool for the V-FUCHS by Wacker et al. was based on a US population, whereas our study consisted of a German cohort. There might be differences especially concerning questions 13 and 14 referring to driving a car. In the age cohort of our study, over 60% of US Americans still drive independently, whereas this number is below 50% in Germany [31, 32]. However, our data showed high rates of patients driving a car.

In conclusion, our study shows a substantial improvement in vision-related quality of life 6 months after DMEK, independent of age, sex, or a possible combination of the procedure with cataract surgery. The equal quality of life increase between surgery on first and second eyes can be taken into account when counseling patients after successful first operation and before indication for the second eye. The fact that postoperative visual acuity and quality of life did not show a strong correlation underlines the rationale for quality of life assessment evaluation, whether via personal doctor-patient-conversation or via structured instruments like the V-FUCHS.

## Supplementary Information

Below is the link to the electronic supplementary material.

Supplementary file1 (PDF 59 KB)
