# Supplementary material for: Descemet membrane endothelial keratoplasty (DMEK) improves vision-related quality of life
Source: Graefes Arch Clin Exp Ophthalmol. 2022 May 25;260(11):3639–45. doi: 10.1007/s00417-022-05711-9 (PMC9581807; doi:10.1007/s00417-022-05711-9)
Supplement: Supplementary file 1 — Supplementary file1 (PDF 59 KB) [file 417_2022_5711_MOESM1_ESM.pdf]

MAYO CLINIC

Visual Function and Corneal Health Status (V-FUCHS)

Form content retained in medical record.
Discard after electronic entry.

(complete fields or place patient label here)

Patient Name *(First, Middle, Last)*	
Birth Date *(mm-dd-yyyy)*	Room Number (if applicable)
Mayo Clinic Number	

BACKUP

Outage Date______________________ Outage Time______________
(mm-dd-yyyy) *(hh:mm 24-hour clock)*

Your Vision in Everyday Life

You are being asked to answer the following questions because we want to understand how your eyesight limits your everyday life. It takes about five minutes to complete the questions.
When you answer the following questions we ask you to think only of the difficulties that your sight may be causing you. Please only consider the impact of your sight, and not other problems, in your ability to perform the following tasks.
If you wear glasses or contact lenses for a particular activity, please answer all of the following questions as though you were wearing your best glasses or contact lenses if necessary.

**How often do you have each of the following difficulties?** For each question, please select the response that corresponds best to your situation (while wearing your best glasses or contact lenses if necessary).	**Never**	**Rarely**	**Sometimes**	**Most of the time**	**All of the time**
During the past month, my eyesight changed over the course of the day.	☐	☐	☐	☐	☐
During the past month, I have had blurred vision that is worst in the morning.	☐	☐	☐	☐	☐
During the past month, I have had trouble with focusing that is worst in the morning.	☐	☐	☐	☐	☐
At night, bright lights look like a starburst.	☐	☐	☐	☐	☐
At night, a bright circle (halo) appears to surround lights, such as street lights.	☐	☐	☐	☐	☐
Overall, fine details are becoming harder to see, for example, leaves on trees.	☐	☐	☐	☐	☐
During the past month, my vision interfered with my daily activities.	☐	☐	☐	☐	☐

**Because of your eyesight, how much difficulty do you have?** For each question, please select the response that corresponds best to your situation (while wearing your best glasses or contact lenses if necessary).	**No difficulty**	**A little**	**Moderate**	**A lot**	**Extreme difficulty**
Reading ordinary print on paper?	☐	☐	☐	☐	☐
Reading text on a screen?	☐	☐	☐	☐	☐
Doing work or hobbies that require you to see well up close?	☐	☐	☐	☐	☐
Reading text on medicine bottles and package inserts?	☐	☐	☐	☐	☐
Seeing the prices of items when shopping?	☐	☐	☐	☐	☐
Seeing what is ahead of you when you enter from daylight into a shady area, such as entering into a parking ramp?	☐	☐	☐	☐	☐
Seeing what is ahead of you when an oncoming car has headlights on at night?	☐	☐	☐	☐	☐
Seeing what is ahead of you when the sun is low during sunrise or sunset?	☐	☐	☐	☐	☐

Wacker K, Baratz KH, Bourne WM, Patel SV. Patient-Reported Visual Disability in Fuchs Endothelial Corneal Dystrophy Measured by the Visual Function and Corneal Health Status Instrument. Ophthalmology 2018;125(12):1854-1861.

©2019 Mayo Foundation for Medical Education and Research
